# Systematic changes of the static upper body posture with a symmetric occlusion condition

**DOI:** 10.1186/s12891-020-03655-x

**Published:** 2020-09-26

**Authors:** C. Maurer-Grubinger, I. Avaniadi, F. Adjami, W. Christian, C. Doerry, V. Fay, V. Fisch, A. Gerez, J. Goecke, U. Kaya, J. Keller, D. Krüger, J. Pflaum, L. Porsch, C. Wischnewski, B. Scharnweber, P. Sosnov, G. Oremek, D. A. Groneberg, D. Ohlendorf

**Affiliations:** 1 Institute of Occupational Medicine, Social Medicine and Environmental Medicine, Goethe-University Frankfurt/Main, Theodor-Stern-Kai 7, Building 9A, 60590 Frankfurt/Main, Germany; 2 Department of Orthodontics, School of dentistry “Carolinum”, Goethe-University Frankfurt am Main, Theodor-Stern-Kai 7, Building 29, 60590 Frankfurt/Main, Germany

**Keywords:** Upper body posture, Healthy adults, Videorasterstereography, Blocked occlusion

## Abstract

**Background:**

Temporary occlusal changes and their influence on the upper body statics are still controversially discussed. Furthermore, concrete statements on whether age- or gender-specific differences in neurophysiological reactions exist are missing. Therefore, it is the aim of this study to evaluate the immediate effects of a symmetrical occlusion blocking on the upper body posture. These effects shall be investigated for both genders and for a larger age range.

**Methods:**

In this study, 800 (407f/393 m) subjects volunteered aged from 21 to 60 years. Both genders were divided into four age groups according to decades. The three-dimensional upper body posture was measured by using the rasterstereography (ABW-Bodymapper). The habitual static posture was measured in two dental occlusion conditions (a) in rest position and (b) symmetrical blocking in the bicuspid region by cotton rolls.

**Results:**

A significant reduction of the trunk length (0.72 mm; *p* <  0.001), an increase of the lumbar (0.30°; *p* <  0.001) and the thoracic bending angle (0.14°; *p* = 0.001), a reduction of the spinal forward decline (0.16°; p <  0.001) and a reduction of the scapular distance (0.36 mm; p = 0.001) was found. Gender-specific reactions can only be recorded in scapular distance, in that regard men reduce this distance while over all age groups women did not show a significant change.

**Discussion:**

Slight gender- and age-independent reactions due to a symmetric occlusion blockade are shown: A gender independent reaction of the spinal related variables in the sagittal plane (thoracic and lumbar flexion angle, trunk length, spinal forward decline). In addition, a gender specific change of the shoulder blade distance could be observed, where men reduced the distance while female did not show a change. However, since these reactions are of a minimum amount, it can be concluded that neurophysiological compensation mechanisms work equally well regardless of age and sex, and the upper body posture of healthy people changes only very slightly due to a temporarily symmetrical altered bite position.

## Background

While the temporal bone is rigidly attached to the other bones of the skull, the mandible is only attached through several muscles to the clavicle and the upper edge of the trunk. In addition, the position of the mandible to the temporal bone does influence the respiratory outcomes [1]. Subject will optimize head position to maximize air flux [1, 2]. The changes of mandible and head positions might be transferred through muscle chains to the upper body [3].

Until now the relationship between the temporomandibular system and the static body posture is controversially discussed [4–15]. Researchers could link pathological postures to specific occlusion characteristics, especially in children and adolescents, respectively. In children with idiopathic scoliosis asymmetric features of malocclusion were found compared with a random control population [6]. A recent review concluded, that there might be an increased prevalence of occlusal dysfunction in patients with known spinal deformity [5]. Yet, there might be a high risk of bias as analysed studies had mainly cohorts of consecutive patients, neither a priori sample size calculation nor well described classifications for the diagnosis of scoliosis, kyphosis and Scheuermann’s disease. Looking on the effects that the temporomandibular system might have on the posture, Korbmacher et al. [7] reported that children with unilateral crossbite might have an higher occurrence of orthopaedic disturbances. Contrary, no significant correlation could be observed between different overjet groups and the upper body posture [8]. In this study differences in the lordotic angle and pelvic inclination between male and female young adults were reported, suggesting that, female and male subjects have different correlations. Some studies investigated the influence of treatments of the temporomandibular system and the change in upper body posture: Parrini et al. [16] reported that the kyphotic angle, the upper thoracic inclination and the pelvic inclination increased throughout aligner therapy. Contrary, an occlusion interference with a 0 to 2 mm thick class composite in healthy adults did not show a change in upper body posture during a 14 day follow-up period [9]. In both studies only a small number of subjects (15 and 12, respectively) was investigated.

To understand the link between specific occlusion condition and body posture better, investigations were initiated on immediate effects of different occlusion conditions of the body posture [4, 9–11, 16–27]. In a pilot study, März et al. [10] could demonstrate that the fleche lombaire and the Kyphotic angle changed slightly with a symmetrical occlusal interference with cotton rolls compared to habitual bite position. Due to a high standard deviation, no compelling conclusion could be drawn, and the researcher suggested a similar protocol with a higher number of subjects. In summary, there might be a relationship between occlusion conditions and body posture.

The shortcoming of previous literature was the small sample size of the studies and the mainly young subjects evaluated. Further investigations should include a larger cohort where the sample size can be estimated from the previous literature. Optimally, it includes a larger range of the population both in terms of age and in terms of gender.

Gender influences a variety of biological functions like the pain threshold, hormone balance, connective tissue and muscularity [28–31]. The body composition of the two genders is distinct, men have wider shoulders and smaller pelves [32–34], the kyphosis angle is larger than the lordosis angle for male subjects [32–34] and the lordosis angle of the male subjects is about 8° smaller than in female subjects [32–35].

Age-specific effects on posture must also be taken into account. Besides the diverging sociological and psychological aging processes, the body also changes from a physiological point of view in the course of a lifetime [36]. Occasionally, if the body weight remains the same, the body proportions may also change, possibly also the posture [37, 38]. With increasing age, muscle and lean body mass decrease and with increasing fat content the water content of the body decreases [39]. A lower muscle mass also implies a lower muscle strength, which changes posture and body tension when standing and sitting [37, 38]. An increased distribution of fat is mainly found in the abdomen area. The changed body composition results in a decreased energy consumption with at least the same amount of required nutrients [36]. Furthermore, less physical activity could further promote muscle loss and thus reduce the energy requirement for the physical activity level. Furthermore, the reduction of bone substance promotes osteoporosis [36]. The body height decrease with increasing age by lower water content of the disci intervertebrae [36]. The prevalence of temporomandibular disorder (TMD) is also not age independent [40].

Therefore, it is important, to include both genders and subjects in a wide age range in order to draw conclusions for the male and female population as well as for young and elderly persons. To measure the expected large sample size, a cost efficient measurement tool has to be used. The rasterstereography is a non-invasive surface scan that can be used to visualize a 3D surface [41–45]. The clear benefit to a 2 dimensional photography [46] or video analysis [47, 48] is the depth information calculated from the projected lines onto a curvature. With progress in image reconstruction algorithms, surface parameters of the upper body can be calculated [32, 34, 41, 49]. Obviously, it cannot measure below the surface, therefore a quantification of spinal parameters is not possible. However, with the addition of markers placed on anatomical landmarks a high intraclass correlation coefficients and good Cornbach’s Alpha values for intra and interday reliability for all spine parameters can be achieved [50–53]. Furthermore, a good inter-tester reliability of 0.979 is reported [45]. Due to the reliable measurement of several parameters of the upper and lower back, some studies have investigated the immediate ad-hoc effects or used the system for correlation analysis [6–10, 16].

Based on the previous discussion the aim of this study is to evaluate the immediate effects of a specific occlusion condition on the upper body posture. These effects shall be investigated for both genders and for a larger age range. A common occlusion condition is the symmetric occlusion in the bicuspid area with paper stripes. This method is known as Meersseman test [54] and often carried out slightly modified with cotton rolls [55, 56]. This test is also used in the field to evaluate misalignments of the bite positions. Based on the previous discussion the main hypothesis investigated in this study is that a symmetric occlusion introduces immediate effects on the upper body posture independent of age and gender compared to the habitual occlusion.

## Methods

### Subjects

Based on the study of März et al. [10] the detectable change with the occlusion condition was in the range of half the standard deviation. With a chosen power of 0.8 and 23 variables in the 4 age and 2 sex groups (Bonferroni correction of multicomparison) that were of interest, this resulted in a minimal sample size of 87 per age and gender range. Therefore, in this study we aimed for 100 subjects per age and sex group.

In this study, 800 (407f/393 m) subjects volunteered. Subjects ranged from 21 to 60 years in age for both genders and were divided into four age groups with equal amounts (Table 1). Only one group had slightly fewer subjects (male between 41 and 50).

**Table 1:** Biometric distribution of the investigated subjects

Sex	Age group	Age Group number	n	Height [m]	Weight [kg]	BMI [kg/m^2^]
female	21–60		407	1.67 +/− 0.06	66.39 +/− 12.68	23.8 +/− 4.6
female	21–30	1	106	1.69 +/− 0.06	60.28 +/− 7.85	21.1 +/− 2.6
female	31–40	2	105	1.66 +/− 0.06	67.03 +/− 13.39	24.2 +/− 4.6
female	41–50	3	98	1.66 +/− 0.06	69.46 +/− 14.28	25.2 +/− 5.0
female	51–60	4	98	1.66 +/− 0.06	69.22 +/− 12.31	25.0 +/− 4.6
male	21–60		393	1.80 +/− 0.07	84.95 +/− 13.59	26.1 +/− 3.6
male	21–30	1	92	1.81 +/− 0.07	76.98 +/− 9.98	23.5 +/− 2.1
male	31–40	2	101	1.80 +/− 0.07	86.36 +/− 11.58	26.7 +/− 3.3
male	41–50	3	100	1.81 +/− 0.08	87.76 +/− 14.57	26.8 +/− 3.6
Male	51–60	4	100	1.80 +/− 0.08	88.07 +/− 14.62	27.0 +/− 3.9

According to the World Health Organization (WHO) classification [57], all women are of normal weight, while men are pre-obese. With regard to the age group analysis of the women, they are normal weight up to the age of 40 years and from then on pre-adipose (25.2 and 25.0 kg/m^2^). In contrast, men are normal weight up to the age of 30 years and from then on pre-obese with 26.7, 26.8 and 27 kg/m^2^ respectively.

All subjects were subjectively healthy without previous postural diseases and/or temporo-mandibular disorders. Before the study conducted, each participant had to sign a written consent and complete a medical history form and anamnesis questionnaire (Centre for Dental, Oral and Maxillofacial Medicine of the Goethe University Frankfurt am Main [58]). The latter included questions on general diseases such as osteoporosis, diabetes mellitus, pain in the joints in general, noises in the ears as well as complaints in the temporomandibular joint. The test persons were also asked about possible accidents in the mouth, jaw and face areas and in the musculoskeletal system.

The study was in accordance with the 1964 Helsinki Declaration and its later amendments and was approved by the local medical ethics committee of the Faculty of Medical Science, Goethe University Frankfurt, Germany (approval No. 303/16).

### Measurement system

For the measurement of the three-dimensional upper body posture, the contactless, light-optical back scanner “ABW-BodyMapper” (ABW GmbH, Frickenhausen/Germany) was used. The associated procedure is called videorasterstereography. The depth resolution of the generated resultant image is 1/100 mm and the maximum image frequency is 50 frames/sec. Measurement errors during recording should be < 1 mm and the measurement accuracy was specified as less than 0.5 mm according to the manufacturer. In order to achieve optimum measurement of the back surface, for each test person six anatomical landmarks (light-reflecting markers of 1 cm diameter) were necessary. The ABW-BodyMapper has already been used successfully in studies to determine representative standard values for young men (18–35 years) [32] and middle-aged men [41–50] [33] as well as women (21–30 years) [59]. Formula or algorithm used to calculate the evaluation parameters are published by Ohlendorf et al. [33].

### Measure protocol

The test persons stood barefoot in a habitual posture, approximately 90 cm in front of the back scanner, with their arms hung loosely, looking horizontally at the opposite wall. In order to obtain reproducible values, firstly three repeated measurements with habitual occlusion situation were performed within 2 min and afterwards with a symmetrical blocked occlusion in the area of the bicuspid with cotton rolls.

### Evaluation of parameters

**Fig. 1:**
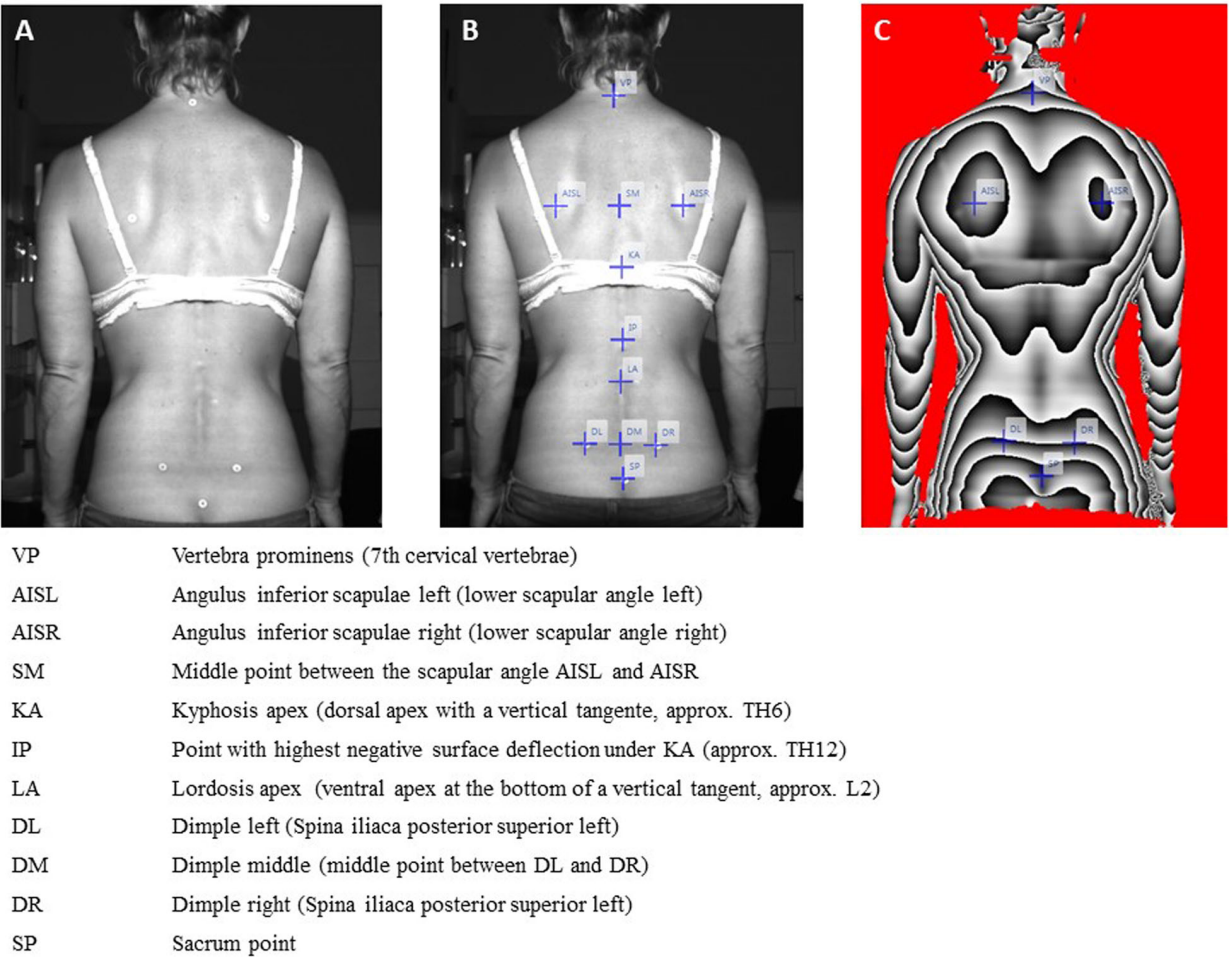
Figure. 1 shows the markers set on the spine (**a**), their names and additional positions required for the calculation of all evaluation parameters (**b**) as well as a depth image of the video raster stereography in which the six glued markers and their names can be seen. All abbreviations are explained at the bottom of the figure

**Fig. 2:**
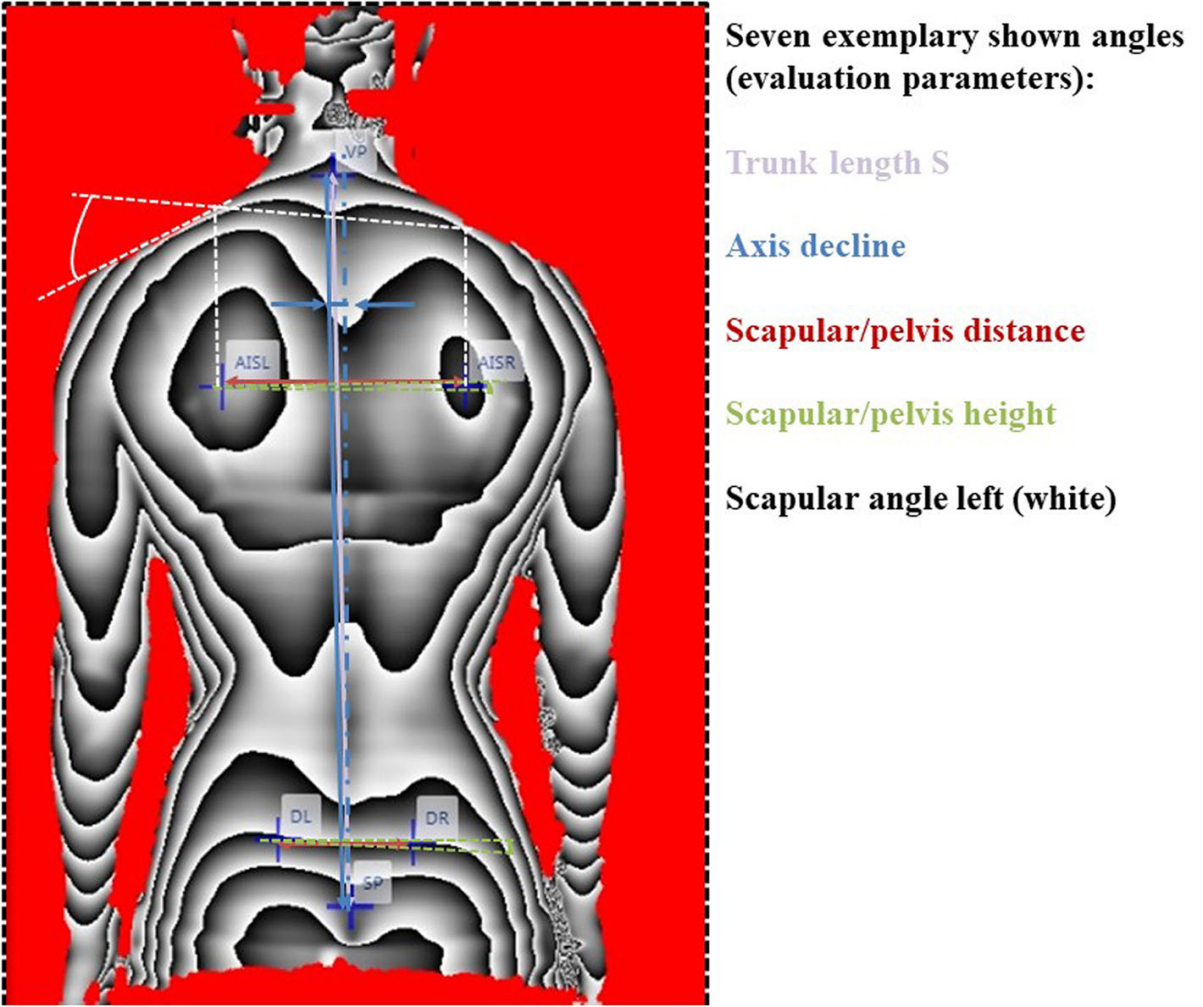
Seven exemplary shown angles (evaluation parameters) in a videorasterstereography image

All parameters were divided into three categories according to the anatomical topography: (a) the markers of the spinal column variables ranged from the 7th cervical vertebra to the rima ani, (b) the shoulder variables enclosed markers on the shoulder blade, (c) the pelvic variables were derived from the marker positions on the left and right SIPS (spina ilica posterior superior). The precise placement of the six landmarks is illustrated in the study protocol of Ohlendorf et al. [60] and all the formulas for calculating each evaluation parameter are published in Ohlendorf et al. [33]. Figure 1 shows the markers set on the spine (A), their names and additional calculated positions required for the calculation of all evaluation parameters (B) as well as a depth image of the videorasterstereography in which the six glued markers and their names can be seen. All abbreviations are explained at the bottom of the figure. In addition, Fig. 2 illustrates seven exemplary angles (evaluation parameters) in the videorasterstereography image.

The definitions of the parameters are specified and defined accordingly by the manufacturer and are therefore adopted in the following evaluations.

- The 13 variables for the spinal column are: trunk length D [mm], trunk length S [mm], sagittal trunk decline [°], frontal trunk decline [°], axis decline [°], thoracic bending angle [°], lumbar bending angle [°], the standard deviation (SD) of lateral deviation [°], maximal lateral deviation [°], SD of rotation [°], maximum of rotation [°], kyphosis angle [°], lordosis angle [°].
- The five variables of the shoulder are: scapular distance [mm], scapular height [°], scapular rotation [°], scapular angle left [°], scapular angle right [°].
- The five variables of the pelvic are: pelvis distance [mm], pelvis tilt [°], pelvis tilt [mm], pelvis torsion [°], pelvis rotation [°].

### Statistics

Statistical analysis was done in matlab (Version 2018a). Significance was tested using a manova. Prior to the manova data were inspected. Normal distribution was tested with the Lilliefor test [61]. In case of none-normal distribution, normal distribution was calculated through the rank distribution of the data. This was done for all variables. The difference of the rank distribution of all variables was calculated and subjected to a manova. The Wilk test was used to evaluate the multiple comparisons. The response of the model were the difference between the two conditions for all 23 variables. The independent factors were sex, age group and the interaction of sex and age group, the dependent variable was the difference between the two conditions. (Wilkinson notation: sex + age group + sex:age group). In addition to the manova the dependency between (body mass index) BMI and variables was checked with a linear regression analysis. A post hoc test was performed for significant values. Either the Students T-tests or an anova was applied, depending if two or more comparisons had to be performed. For the post hoc tests a Bonferroni correction was applied. Data were presented as the original data (not rank transformed) for a better understanding. Significance level was set to 0.05.

## Results

Only three variables were normal distributed within all sub groups: Trunk length S, Scapular distance and Kyphosis angle. Therefore, all data were transformed to a normal distribution. The manova test on the difference between the habitual and the cotton roll conditions revealed significant results for at least one variable across the whole sample (F(22,775) = 1,78 (*p* = 0.015)), and between the two sex groups (F(22,775) = 1,67 (*p* = 0.028)) (Table 2). Over all gender and age groups, at least one variable showed a significant result (first line, within variable between intercept evaluates if the difference between the conditions differs from zero). There is also a significant difference between one variable and the sex (p = 0.028). Within the age group and the interaction between age groups and sex did not show a significance.

**Table 2:** Result of the manova. Differences could be observed within at least one variable over all subjects and between the sex group

Within	Between	Statistic	Value	F	R^2^	df1	df2	*P* value
variable	(Intercept)	Wilks	0.95	1.78	0.048	22	775	0.015
variable	sex	Wilks	0.95	1.67	0.045	22	775	0.028
variable	Agegroup	Wilks	0.96	1.49	0.041	22	775	0.069
variable	sex:agegroup	Wilks	0.97	1.11	0.031	22	775	0.325

**Fig. 3:**
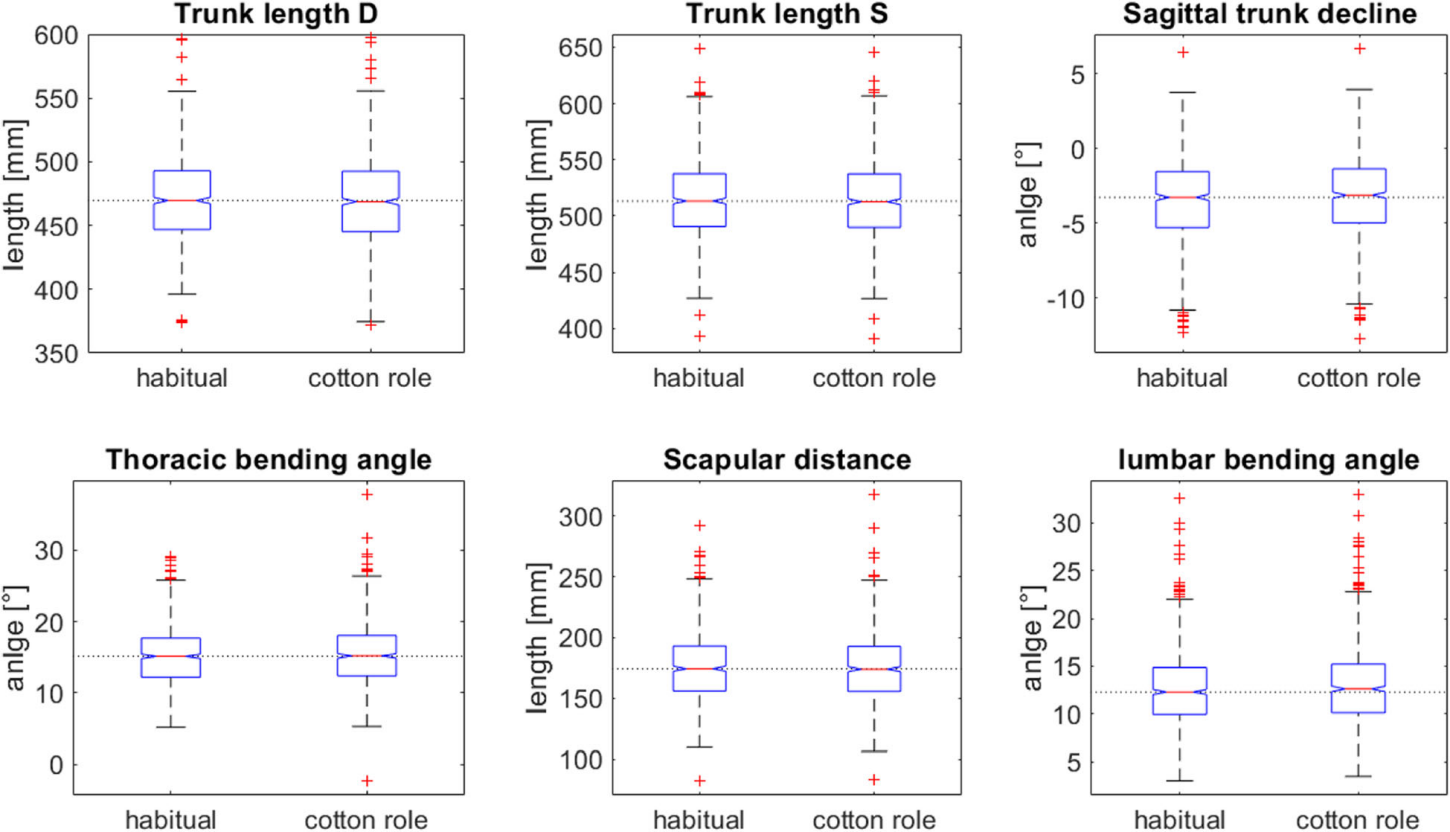
Box plots of the significant variables

A student’s t test showed that over all subjects the trunk length D and S, the sagittal trunk decline, the thoracic bending angle, the scapular distance and the lumbar bending angle showed significant differences Table 3. The mean and quartiles were calculated from the original distribution and the statistics were calculated from the normal rank transformed. The very small but systematic changes are displayed in Fig. 3. The trunk length was reduced by the occlusion block for 0.72 mm and 0.87 mm (for the trunk length D and the trunk length S, respectively). The sagittal trunk decline was reduced by 0,16° resulting in a slightly more upright position. The thoracic bending angle is increased by 0.14° and the lumbar bending angle is increased by 0.3° with the cotton roll, and the scapular distance is reduced by 0.36 mm with the cotton roll.

**Table 3:** Descriptive statistics of all the variables over the whole sample size.* after the variable indicates a significant difference. For all variables the degree of freedom is df = 799

Variable names	Habitual			Cotton role			Difference cotton role - habitual			T value	*P* value
	Median	1st quantile	3rd quantile	Median	1st quantile	3rd quantile	Median	1st quantile	3rd quantile		
Spinal column:											
Trunk length D [mm]*	**470**	**447**	**493**	**469**	**445**	**493**	**−0.72**	**− 2.09**	**0.55**	**5.1**	**< 0.001**
Trunk length S [mm]*	**513**	**490**	**537**	**513**	**490**	**537**	**−0.87**	**−2.32**	**0.66**	**5.0**	**< 0.001**
Sagittal trunk decline [°]*	**−3.28**	**−5.30**	**−1.55**	**− 3.13**	**− 4.99**	**− 1.37**	**0.16**	**−0.33**	**0.73**	**−6.6**	**< 0.001**
frontal trunk decline [°]	−0.16	− 1.00	0.67	− 0.16	− 1.00	0.68	− 0.01	− 0.30	0.24	1.5	0.144
Axis decline [°]	−0.67	−2.00	0.91	−0.64	−2.00	1.04	0.01	−0.33	0.33	− 0.3	0.757
Thoracic bending angle [°]*	**15.1**	**12.2**	**17.7**	**15.2**	**12.4**	**18.1**	**0.14**	**−0.50**	**0.87**	**−3.4**	**0.001**
lumbar bending angle [°]*	**12.3**	**9.9**	**14.9**	**12.6**	**10.1**	**15.2**	**0.30**	**−0.16**	**0.89**	**−4.7**	**< 0.001**
SD of lateral deviation[°]	3.81	2.65	5.33	3.94	2.65	5.54	0.06	−0.49	0.63	−1.3	0.178
Maximal lateral deviation [°]	−4.27	−7.79	3.58	−4.57	−7.99	4.21	−0.03	−1.31	1.13	0.4	0.661
SD of rotation [°]	3.90	2.74	5.51	4.02	2.71	5.76	0.07	−0.62	0.79	−2.3	0.019
Maximum of rotation [°]	4.30	−6.62	8.66	4.56	−6.99	8.75	−0.01	−2.23	2.11	1.0	0.331
Kyphosis angle [°]	53.3	45.6	61.7	53.4	45.8	62.8	0.67	−1.18	2.59	−2.7	0.006
Lordosis angle [°]	38.4	30.7	49.6	38.8	31.2	49.6	0.40	− 0.73	1.60	−1.5	0.135
Shoulder variables											
Scapular distance [mm]*	**174**	**156**	**193**	**174**	**156**	**193**	**−0.36**	**−2.25**	**1.12**	**3.5**	**0.001**
Scapular height [°]	−1.99	−6.57	3.43	−2.08	−6.87	3.08	−0.12	−1.28	1.04	2.6	0.010
Scapular rotation [°]	1.28	−0.93	3.43	1.24	−1.00	3.62	0.00	−0.93	0.91	0.1	0.949
Scapular angle left [°]	26.7	23.2	30.9	27.0	23.5	31.0	0.23	−1.15	1.47	−1.9	0.054
Scapular angle right [°]	28.4	24.7	32.7	29.1	25.1	33.0	0.33	−1.51	2.52	−2.4	0.015
Pelvic variables											
Pelvis distance [mm]	98.0	88.7	109.2	97.7	88.7	109.1	−0.01	− 0.31	0.24	0.8	0.398
Pelvis tilt [°]	−0.42	−1.72	1.02	−0.35	−1.72	1.02	0.01	−0.20	0.27	−1.3	0.185
Pelvis tilt [mm]	−0.72	−3.00	1.85	−0.57	−2.96	1.87	0.02	−0.34	0.43	−1.2	0.221
Pelvis torsion [°]	0.36	−2.98	3.68	0.34	−2.81	3.66	−0.01	−0.95	1.00	−1.1	0.286
Pelvis rotation [°]	0.50	−1.96	2.94	0.47	−2.07	3.16	−0.09	−0.98	0.85	0.9	0.388

Between the two genders, only one variable, the scapular distance, showed a significant result. The mean difference of the scapular distance between the cotton roll and the habitual condition showed an increase of the distance by 0.08 mm for female and a reduction of 0.96 mm for the male (Table 4). The difference between the two gender was significant with: t(798) = − 4.64 (*p* <  0.001). Therefore, the overall change of the scapular distance (Table 3) resulted from the male population alone.

**Table 4:** Changes between the two gender groups for the scapular distance between the habitual and the cotton role bite condition

Age Group	Gender	Age range	Variable	habitual			Cotton role			Cotton role – habitual		
				Median	1st quartile	3rd quartile	Median	1st quartile	3rd quartile	Median	1st quartile	3rd quartile
**Overall**	Female*	**21–60**	**Scapular distance**	**161.3**	**145.1**	**177.4**	**160.9**	**145.2**	**177.5**	**0.08**	**−1.44**	**1.66**
1	Female	21–30	Scapular distance	149.8	138.3	163.4	151.8	139.8	163.7	0.85	−1.02	2.83
2	Female	31–40	Scapular distance	158.0	141.4	169.3	156.7	141.7	167.4	−0.05	−1.53	1.35
3	Female	41–50	Scapular distance	176.6	161.1	188.4	177.4	162.0	188.3	0.02	−1.44	1.32
4	Female	51–60	Scapular distance	164.0	150.7	179.7	164.3	149.9	178.9	−0.31	−1.64	1.06
**Overall**	Male*	**21–60**	**Scapular distance**	188.1	171.3	206.0	186.7	170.8	204.7	−0.96	−3.01	0.65
1	Male	21–30	Scapular distance	177.7	162.0	192.3	176.7	160.7	192.8	−1.33	−3.33	0.50
2	Male	31–40	Scapular distance	185.1	166.3	203.4	186.2	166.2	202.7	−0.54	−1.68	0.91
3	Male	41–50	Scapular distance	187.5	174.1	213.2	188.0	173.2	212.2	−1.11	−3.69	0.32
4	male	51–60	Scapular distance	198.4	184.7	212.7	196.8	182.0	212.2	−1.53	−4.09	0.68

While the difference between the whole population of the females and the males was significant based on the Manova, the age groups did not show a significance due to the multiple comparisons.

A full list of the t-tests is also presented (Table 5). The MANOVA revealed no significance between the individual age groups. Therefore, no anova between the age groups was performed on the different variables.

**Table 5:** Test statistic for the comparison of all variables by gender. An * asterix behind the variable name indicates a significant result. The first column is the variable name, the second the median of the difference of the female and male subjects, followed by the confidence interval, the t-value from the student’s t-test, the estimated standard deviation and the probability based on the degree of freedom of 798

Variable	Median of the difference		Confidence interval		t value	estimated standard deviation	*p* value
	Female	Male	Lower	Upper			
Spinal column:							
Trunk length D [mm]	−0.71	−0.74	−0.91	0.36	−0.85	4.57	0.396
Trunk length S [mm]	−0.83	−0.92	− 0.58	0.75	0.25	4.80	0.805
Sagittal trunk decline [°]	0.12	0.24	−0.23	0.04	−1.43	0.96	0.153
frontal trunk decline [°]	−0.05	0.00	−0.05	0.07	0.32	0.46	0.747
Axis decline [°]	0.02	0.00	−0.09	0.11	0.20	0.73	0.839
Thoracic bending angle [°]	0.12	0.16	−0.31	0.17	−0.59	1.71	0.554
lumbar bending angle [°]	0.27	0.33	−0.37	0.13	−0.96	1.80	0.339
SD of lateral deviation [°]	0.09	0.02	−0.21	0.13	−0.44	1.25	0.660
Maximal lateral deviation [°]	−0.01	−0.09	− 0.23	0.79	1.08	3.66	0.280
SD of rotation [°]	0.01	0.10	−0.36	0.09	−1.19	1.62	0.233
Maximum of rotation [°]	−0.22	0.04	−2.24	0.10	−1.79	8.45	0.074
Kyphosis angle [°]	0.71	0.67	−1.35	0.51	−0.88	6.69	0.377
Lordosis angle [°]	0.30	0.49	−1.34	0.30	−1.25	5.91	0.211
Shoulder variables							
Scapular distance [mm]*	**0.08**	**−0.96**	**0.62**	**1.91**	**3.87**	**4.64**	**< 0.001**
Scapular height [°]	−0.20	0.00	−0.36	0.33	−0.07	2.51	0.942
Scapular rotation [°]	−0.07	0.00	−0.39	0.12	−1.04	1.83	0.299
Scapular angle left [°]	0.08	0.30	−0.91	0.83	−0.10	6.27	0.924
Scapular angle right [°]	0.17	0.43	−2.10	0.38	−1.37	8.92	0.172
Pelvic variables							
Pelvis distance [mm]	−0.02	0.00	−0.29	0.34	0.16	2.30	0.876
Pelvis tilt [°]	0.04	0.00	−0.08	0.10	0.19	0.64	0.851
Pelvis tilt [mm]	0.07	0.00	−0.11	0.20	0.56	1.12	0.578
Pelvis torsion [°]	−0.01	0.00	−0.16	0.38	0.80	1.92	0.422
Pelvis rotation [°]	−0.14	− 0.07	−0.38	0.09	−1.22	1.70	0.224

**Fig. 4:**
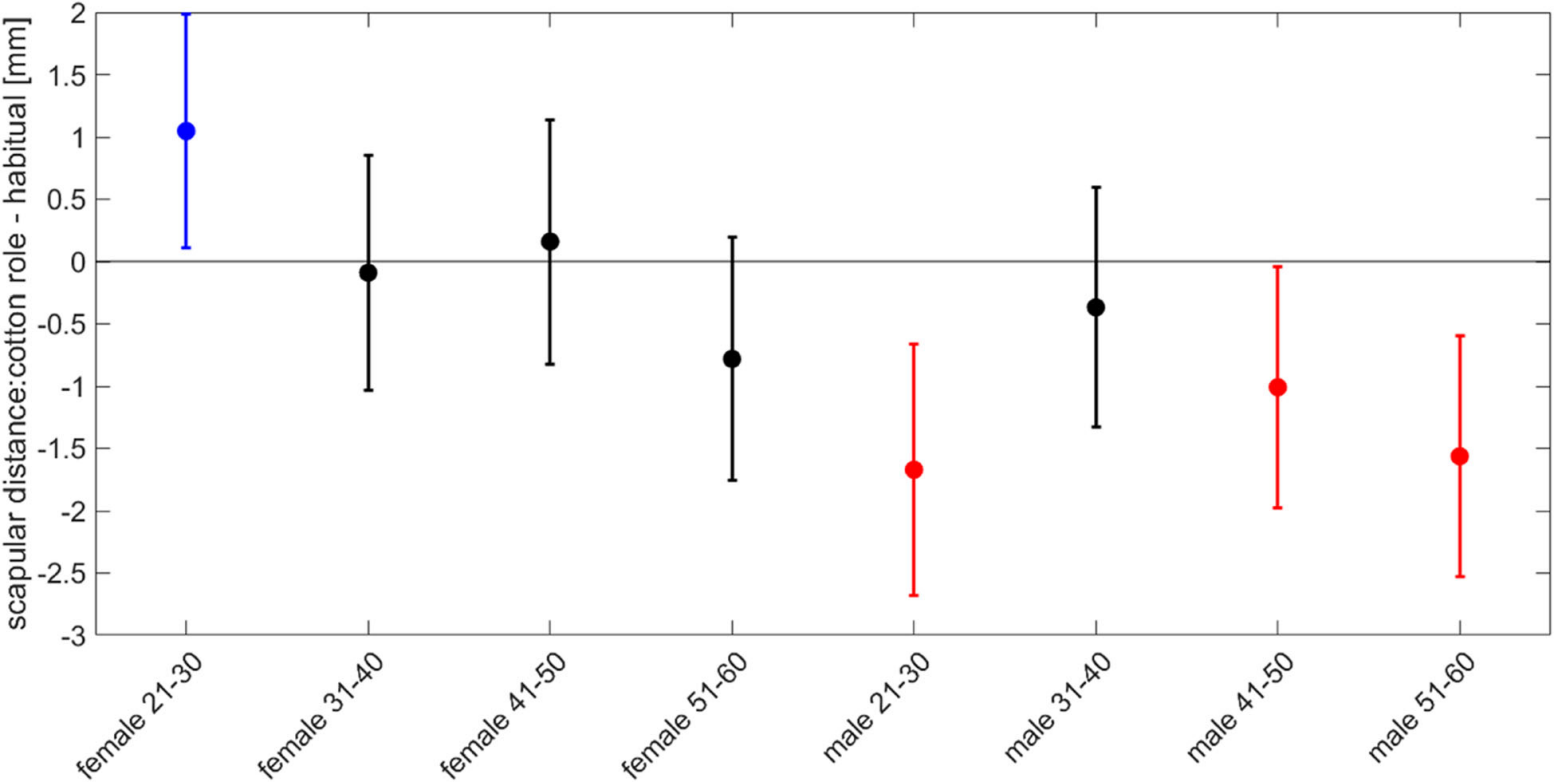
Median and confidence intervals of the scapular change for the 4 different age groups of female and male subjects. The first four bars are the female age groups, the last four are the male age groups. The only significant difference can be found between the youngest female group (blue) and the three male age groups (21–30, 41–50 and 51–60) (all in red)

A closer look into the age and gender groups reveals that there are no significances between the sexes and age groups except the scapula distance. There is a difference between genders which mainly can be tracked back to the young female subjects. The scapular distance of the female subjects aged 31 to 40 years and 41 to 50 years remains unchanged between the two conditions while the young female aged 21 to 30 and the older females aged 51 to 60 years do so. While the scapular distance of the young females increased with the cotton roll by 0.85 mm, the distance of the elderly reduced by 0.31 mm. The reduction of scapular distance by biting on cotton rolls is not only observed in the oldest group of women, but also in three of four male age groups. Only age group 2 (31–40 years) shows no significant difference to the youngest female group (Table 4 and Fig. 4). Although the results alone might be significant, within the current measurement protocol they are not significant because of multiple comparisons.

**Fig. 5:**
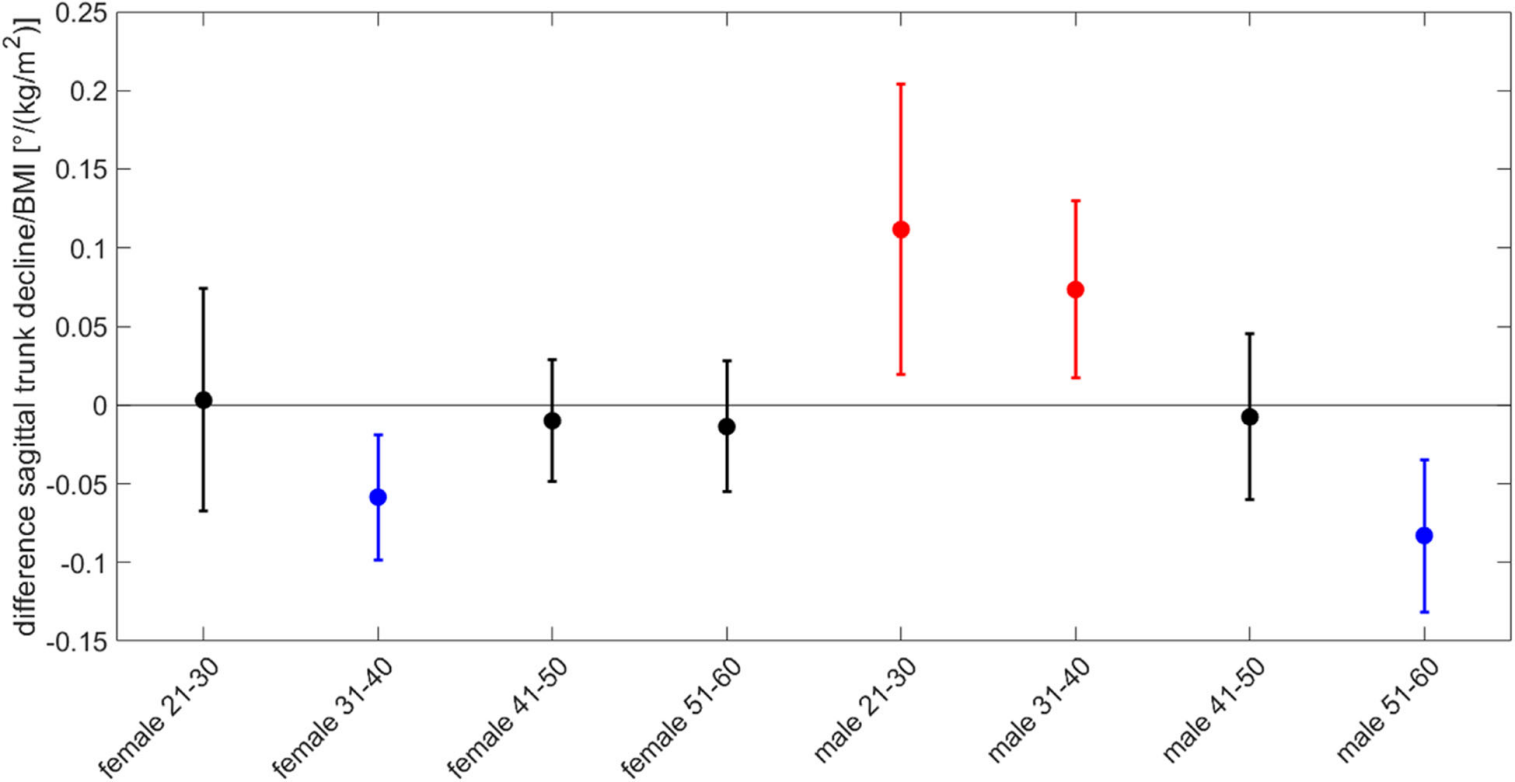
Dependency between difference of sagittal trunk decline and BMI. The slope of the dependency is plotted against the 8 sex and age groups. Median and confidence intervals of the slope between the sagittal trunk decline change and the BMI for the 4 different age groups of female and male subjects is plotted . The first four bars are the female age groups, the last four are the male age groups. Two groups have a significant positive relationship with BMI (male aged 21–30 and male aged 31–40, red) and two groups have a negative relationship between the decline change and BMI (female 31–40 and male 51–60, blue)

Overall the BMI did not have a linear relationship with any of the 23 variables Table 6. The BMI ranges from 16 to 43.1 with a median BMI of 24. At the age group and sex level the difference of the sagittal trunk decline had a group specific difference between the BMI and the variable (Table 6). Two groups have a positive relationship with BMI, the male aged 21–30 and male 31–40 (Fig. 5). With increasing BMI the decline in the sagittal plane increased. With a slope of 0.11 and 0.07 the decline increases by 1° per BMI decade. Two groups have a negative relationship with BMI, the female aged 31–40 and male aged 51–60. Within these two groups the decline reduced with increasing BMI resulting in a more upright position with increasing BMI. The slope is − 0.06 and − 0.85, respectively. The two groups with the positive slope are significant different from the two groups with negative slope.

**Table 6:** Influence of the BMI on the individual variables. An * asterix behind the variable name indicates a significant result. The first column is the variable name, the second the slope between BMI and the variable, followed by the confidence interval, the F-value from the anova for the interaction effect and the corresponding *p*-value

Variable	Slope	Confidence interval		Anova for interaction	
		Lower	Upper	F value	*p* value
Spinal column:					
Trunk length D [mm]	0.007	−0.109	0.123	0.939	0.475
Trunk length S [mm]	0.015	−0.107	0.137	1.027	0.410
Sagittal trunk decline [°] *	**−0.023**	**−0.047**	**0.001**	**4.329**	**< 0.001**
frontal trunk decline [°]	−0.001	−0.013	0.011	0.185	0.988
Axis decline [°]	0.000	−0.019	0.019	0.709	0.664
Thoracic bending angle [°]	−0.011	−0.054	0.032	1.617	0.127
lumbar bending angle [°]	0.008	−0.038	0.054	0.734	0.643
SD of lateral deviation [°]	−0.001	−0.033	0.031	0.466	0.859
Maximal lateral deviation [°]	−0.013	−0.106	0.080	1.788	0.087
SD of rotation [°]	−0.025	−0.066	0.016	0.633	0.729
Maximum of rotation [°]	−0.073	−0.287	0.142	2.387	0.020
Kyphosis angle [°]	0.153	−0.016	0.322	1.719	0.101
Lordosis angle [°]	0.098	−0.052	0.248	1.452	0.181
Shoulder variables					
Scapular distance [mm]	0.071	−0.048	0.189	2.676	0.010
Scapular height [°]	0.026	−0.037	0.090	0.717	0.658
Scapular rotation [°]	0.002	−0.045	0.048	1.231	0.283
Scapular angle left [°]	−0.021	−0.180	0.138	0.433	0.882
Scapular angle right [°]	0.030	−0.197	0.256	1.030	0.409
Pelvic variables					
Pelvis distance [mm]	0.018	−0.040	0.076	0.222	0.980
Pelvis tilt [°]	0.002	−0.014	0.018	0.958	0.461
Pelvis tilt [mm]	0.006	−0.022	0.034	0.799	0.588
Pelvis torsion [°]	0.016	−0.033	0.064	0.835	0.558
Pelvis rotation [°]	0.001	−0.042	0.044	1.036	0.404

## Discussion

The cotton roll induced some changes of the spine related variables in all subjects: a reduction of the trunk length (trunk length D and trunk length S), an increase of the lumbar and the thoracic bending angles, which goes along with the reduction of the trunk length and a reduction in the forward decline of the sagittal bending angle. In addition, there is a reduction of the scapular distance. Therefore, the hypothesis was not rejected for the general outcome of the occlusion condition nor for the gender aspect. However, no significant difference was found between the age groups. Therefore, the hypothesis, that the age groups show significant different changes to a symmetric occlusion condition has to be rejected.

All these changes are small compared to the interquartile distance of the habitual position. Additionally, the interquartile distance of the differences always crosses zero so that not all subjects change in the same manner with the cotton roll applied. Neither changes have been observed in the pelvis region nor any rotational variable has been changed. Based on these findings there are small but significant differences mainly in the sagittal plane of the spine.

The raster stereography measures the surface of the back. The anatomical features are estimated based on these surface images. The quality of the calculations might also depend on the muscle and fat of the back [62]. In order to permit correct statistical inference, it is therefore pivotal that the sample is drawn from a representative population. Within this study the height, weight and BMI for the age groups 31–40, 41–50 and 51–60 falls within 1% of the German population [63–65]. The height of the youngest age group falls as well within 1%. Only the weight is 6% lower than the population mean. We therefore assume that the sample drawn represents the population well enough.

The spinal variables changed independent of age and gender with no statistical significant difference between age and gender. Males and females of all ages showed the same reaction in the spine variables to the cotton roll occlusion condition. The reason for the reduction in trunk length, increase of lumbar and thoracic bending angle and the small change to an upright upper body can only be speculated. It might be that the slight opening of the mouth with a slight lower position of the mandibular relaxes the muscles of the anterior neck relieving the pull onto the clavicular and the cervical spine. So far, this can only be speculated, because neither an electromyography (EMG) nor a tensiomyography of the neck muscles was conducted. EMG activity of the neck has already been conducted [66–68] . Further research is needed to answer the relationship between occlusion condition and neck muscle activity.

The scapular distance showed the only different reaction on the occlusion condition between genders. While it reduced for the male by about 1 mm, it did not show a significant change for the female subjects. An age specific analysis revealed that this might be because the female age group 21–30 increased the scapular distance. Even though this result is not significant because of the multi comparison correction applied, it indicates a different reaction within the female population. Whether the reduction of the scapular distance for the male subjects occures because of a tightening due to a harder bit onto the cotton role, is up for speculation. It is known, that the bite force is different between genders as well [69, 70]. The bite force is highest for severe brachyfacial male individuals being heavy and tall as well as being between 41 and 50 years old [71]. In addition to the bite force, girls have a higher occurrence of TMDs than boys have [72–74].

März et al. [10] has reported an increase of the kyphotic angle and an increase of the fleche lombaire. Within our study the kyphotic angle increased, but the difference to the habitual condition could be estimated by *p* = 0.008 which is above the required Bonferroni corrected *p* value of 0.05/23 = 0.002. Appling the used multi comparison correction might lead into a false rejection. The fleche lombaire was not measured in this study. However, the fleche lombaire is linked to the lumbar bending angle. The lumbar bending angle did increase, and therefore the fleche lombaire should increase as well.

The changes to the cotton roll occlusion were small especially when compared to the population distribution. For instance, the median change is 4% compared to half the interquartile distance of the thoracic bending angle (0.14/2.8 = 0.05) or 2% for the scapular distance (0.36/18 = 0.02). Therefore, the therapeutic relevance is questionable. It can be argued, that a constant change might affect the muscle load on the upper body, however in this case it has to be investigated, if the change persists over a longer period of time. In addition, so far it is not clear what a healthy upper body posture is, and it might be speculated, that due to the high variability of individual subjects it is hard to find conclusive upper body posture norm values [75]. Within this study we have measured 800 subjects. Therefore, the results obtained have a certain statistically strength.

The practical application of these findings are two fold: 1) An unspecific occlusion blockade in an healthy subject will not make a substantial change of the upper body posture. Therefore, this type of intervention is not enough to correct the upper body posture. 2) In the clinical praxis some tests evaluate the function of the TMD based on the symmetric occlusion blockage. The interquartile distance of the changes presented in Table 3 gives the limits such tests have to exceed in order to be clinical relevant. To our knowledge this is the first time, that such a large sample size was examined and also stratified by gender and age. There are some limitations of this study. The occlusion condition was always the second condition of the measurement procedure. It therefore can’t be ruled out, that the observed effect is simple based on a change of the posture during consecutive measurements. A further limitation are the 23 variables measured. In order to reduce the probability of detecting a false positive change a multi comparison correction was applied. This multi comparison resulted in a smaller probability value in order to be certain to have a real effect, but this could introduce a beta error of falsely reject the hypothesis.

In everyday life, the occlusion changes very often for seconds, minutes or even up to hours at different intervals, for example by chewing food, especially solid food, chewing gum or negative habits. All these impulses are absorbed in the temporomandibular system via high-level, multidimensional neurophysiological circuitry and transmitted to the central nervous system (CNS) [55]. There, the information is evaluated and the reaction to it is planned. Due to the frequency and the tendency of these impulses to have a short period of influence, it makes sense for the maintenance of a stable system, if no far-reaching or energy-intensive adjustments of the entire body posture to this new situation are made, but rather temporary and reversible compensation mechanisms take place. Since all subjects are fully grown and, according to subjective statements, are in a long-term symptom-free state, it can be assumed that this state represents a stable equilibrium. Further investigations could determine the strength and duration required to challenge the compensation processes.

Furthermore, this study focused on the measurement of the back. While this is the first approach to see if there are age- or gender-related systematically reactions of the upper body due to a symmetrical occlusion blocking, additional experiments have to be conducted in order to understand the mechanism behind the change of the upper body due to the occlusion condition. So far, there is no consensus in the scientific community of the functional pathway [12]. It is speculated that the change of the mandibular results in a sensory information that is processed in the central nervous system and effects the muscles of the upper body [76]. Another speculation is that due to the change of the opening of the airflow, the head position has to be readjusted, in order to increase or decrease the air tunnel [1]. As the head is controlled via muscles attached to the upper body this would result in a reaction in order to balance the head again. A third theory is, that the lowering of the mandibular relaxes the muscles of the anterior triangle of the neck [68]. This reduced pull on the spine and clavicular might again be transferred to the muscles of the spine.

Regardless of the extent to which these mechanisms now have a theoretic effect, it is obvious that, regardless of age or gender, a symmetrical occlusal blockage in the bicuspid region has little effect on upper body posture. Thus, it is reasonable to assume that the physiological human mechanisms are independent of gender and age, taking individual differences into account. However, this assumption only applies to persons who feel subjectively healthy. To what extent pathologies of different genesis and in different body regions can change these results must be left to further analysis.

## Conclusion

Slight gender- and age-independent reactions due to the occlusion blockade are shown: slightly more pronounced thoracic and lumbar flexion angle in combination with a reduction in trunk length and contraction of the shoulder blades. The latter can be seen more intensively in men. However, since these reactions are of minimum amount, it can be concluded that neurophysiological compensation mechanisms work equally well regardless of age and sex, and the upper body posture of healthy people changes only very slightly due to a temporarily altered bite position.

## Data Availability

The datasets used and/or analyzed during the current study are available from the corresponding author on reasonable request.

## Abbreviations

AISL: Angulus inferior scapulae left
AISR: Angulus inferior scapulae right
BMI: Body mass index
CNS: Central nervous system
DL: Dimple left
DM: Dimple middle
DR: Dimple right
EMG: Electromyography
IP: Point with highest negative surface deflection under KA
KA: Kyphosis apex
SD: Standard deviation
SIPS: Spina ilica posterior superior
SM: Middle point between AISL and AISR
SP: Sacrum point
TMD: Temporomandibular disorder
VP: Vertebra Prominence
WHO: World Health Organization
